# Spectrophotometric Study of Bridging *N*-Donor Ligand-Induced Supramolecular Assembly of Conjugated Zn-Trisporphyrin with a Triphenylamine Core

**DOI:** 10.3390/molecules26164771

**Published:** 2021-08-06

**Authors:** Nirmal K. Shee, Ju-Won Seo, Hee-Joon Kim

**Affiliations:** Department of Applied Chemistry, Kumoh National Institute of Technology, Gumi 39177, Korea; nirmalshee@gmail.com (N.K.S.); juwon1105@gmail.com (J.-W.S.)

**Keywords:** Zn-trisporphyrin conjugate, supramolecular assembly, bridging *N*-donor ligand, double-decker complexes

## Abstract

This article studies the supramolecular assembly behavior of a Zn-trisporphyrin conjugate containing a triphenylamine core (**1**) with bridging *N*-donor ligands using the UV-vis spectrophotometric titration method at micromolar concentrations. Our results show that pyridine, a non-bridging ligand, formed a 3:1 open complex with **1**. The corresponding binding constant was estimated to be (2.7 ± 0.15) × 10^14^ M^−3^. In contrast, bridging ligands, 4,4-bipyridine (BIPY) and 1,3-di(4-pyridyl)propane (DPYP), formed stable 3:2 double-decker complexes with **1** in solution, which collapsed to yield a 3:1 open complex when excess BIPY or DPYP was added. The binding constants for forming BIPY and DPYP double-decker complexes were estimated to be (9.26 ± 0.07) × 10^27^ M^−4^ and (3.62 ± 0.16) × 10^27^ M^−4^, respectively. The UV-vis titration profiles supported the conclusion that the degradation of the 3:2 double-decker **1**∙BIPY complex is less favorable compared to that of **1**∙DPYP. Consequently, the formation of the 3:1 **1**∙DPYP open complex proceeded more readily than that of **1**∙BIPY.

## 1. Introduction

The construction of well-defined nano- and microscopic architectures provides access to outstanding materials [1,2,3] for use in artificial electron transfer [4], photocatalysis [5], sensing [6], molecular recognition [7], and medicinal applications [8]. Porphyrin materials are attractive building blocks for forming self-assembled, highly ordered supramolecular structures [9,10,11]. Porphyrin compounds are important because of their symmetric aromatic structures and excellent electronic and photophysical properties, and are used for catalysis [12,13,14]. Porphyrin molecules self-assemble to form large-scale aggregates with well-defined nanostructures of definite sizes, shapes, and dimensions. Various intermolecular non-covalent forces or interactions, such as hydrogen bonding, hydrophobic or hydrophilic interactions, π-π stacking, ligand coordination, electrostatic interactions, and van der Waals interactions, are responsible for porphyrin self-assembly [15,16,17,18,19]. Various strategies, such as ionic self-assembly [20], re-precipitation methods [21], ligand coordination [22], coordination polymerization [23], and surfactant assistance by either surfactants or amphipathic molecules [24], have been utilized to form porphyrin aggregates. However, covalent metalloporphyrins, such as dendrimers, double-stranded conjugated ladder porphyrins, porphyrin arrays, double-decker porphyrins, cyclic porphyrins, and tetra-porphyrins, readily self-assemble without any external stimuli and form a larger aggregation of porphyrin arrays in solution [25,26,27,28,29,30]. Among the various supramolecular architectures, tris-metalloporphyrins assembled with *N*-donor ligands, such as 1,4-diazabicyclo[2.2.2]octane (DABCO), form sandwich-type coordination cages that are attractive for supramolecular catalysis applications and artificial molecular receptor synthesis [31,32,33].

**Scheme 1:**
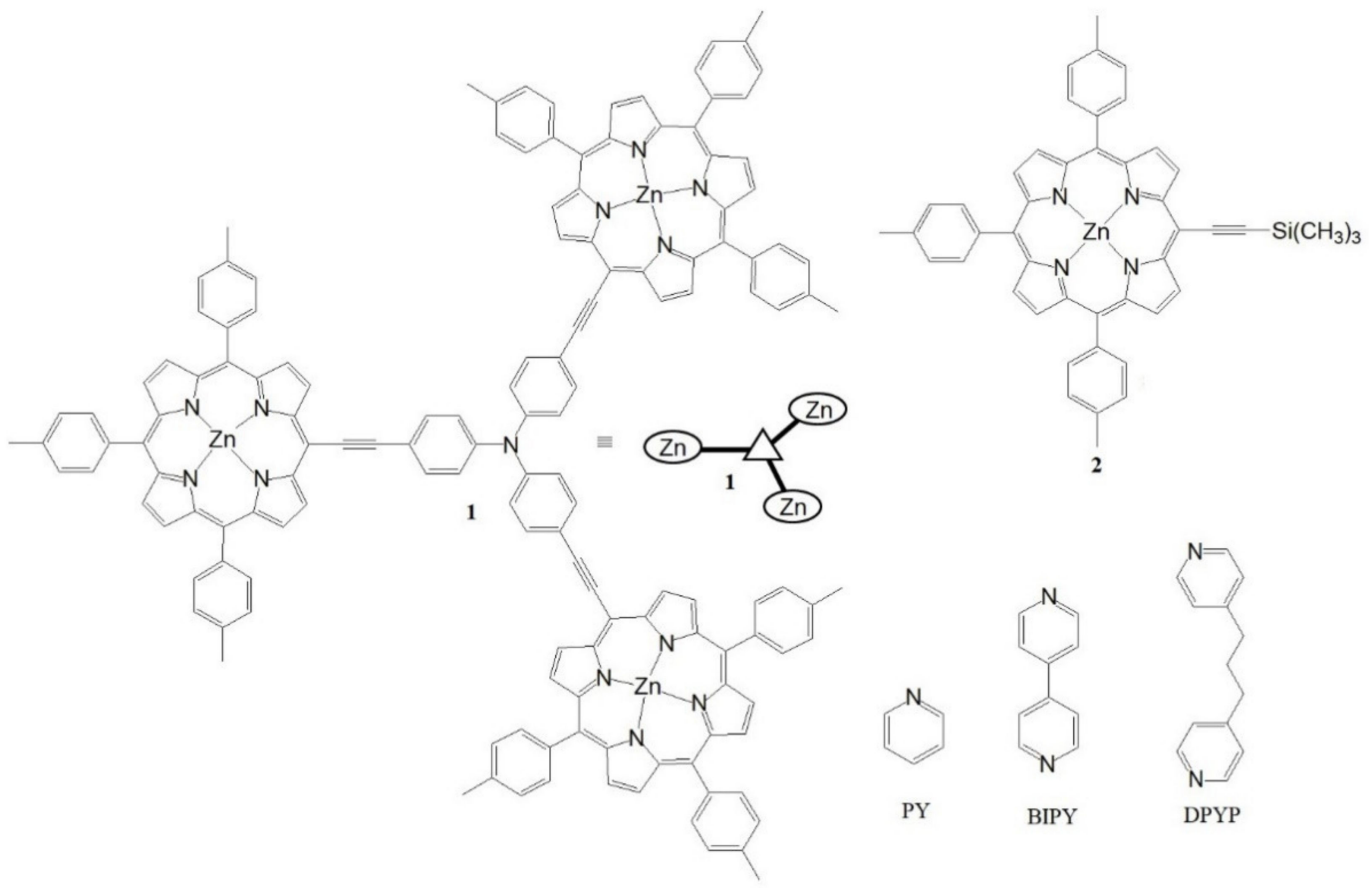
Chemical structures of Zn-porphyrins and *N*-donor ligands used in this study.

We have previously reported the strong two-photon absorption of octupolar trisporphyrin conjugates containing a triphenylamine core (**1**) (Scheme 1) [34]. The optimized geometry obtained using semi-empirical AM1 calculations revealed that the trisporphyrin molecule adopts a propeller-shaped structure. Incorporating the unique *C*_3_-symmetrical structure of conjugated porphyrins, we expected to construct an interesting architecture via supramolecular assembly within this system. In this report, we describe the spectrophotometric study of the supramolecular assembly of Zn-trisporphyrin induced by coordination with bridging *N*-donor ligands. We chose 4,4-bipyridine (BIPY, a rigid ligand) and 1,3-di(4-pyridyl)propane (DPYP, a flexible ligand) as the bridging *N*-donor ligands for the present study.

## 2. Results and Discussion

UV-vis spectroscopy was applied to evaluate the binding properties of Zn-trisporphyrin **1** (1 × 10^−6^ M) with bridging *N*-donor ligands in toluene at room temperature. For a comparative study, we used monomeric Zn-porphyrin **2** ([5,10,15-tri-(*p*-tolyl)-20-trimethylsilylethynylporphyrinato]Zn(II), see Scheme 1) as a reference model and performed supramolecular assembly studies under similar conditions.

**Scheme 2:**
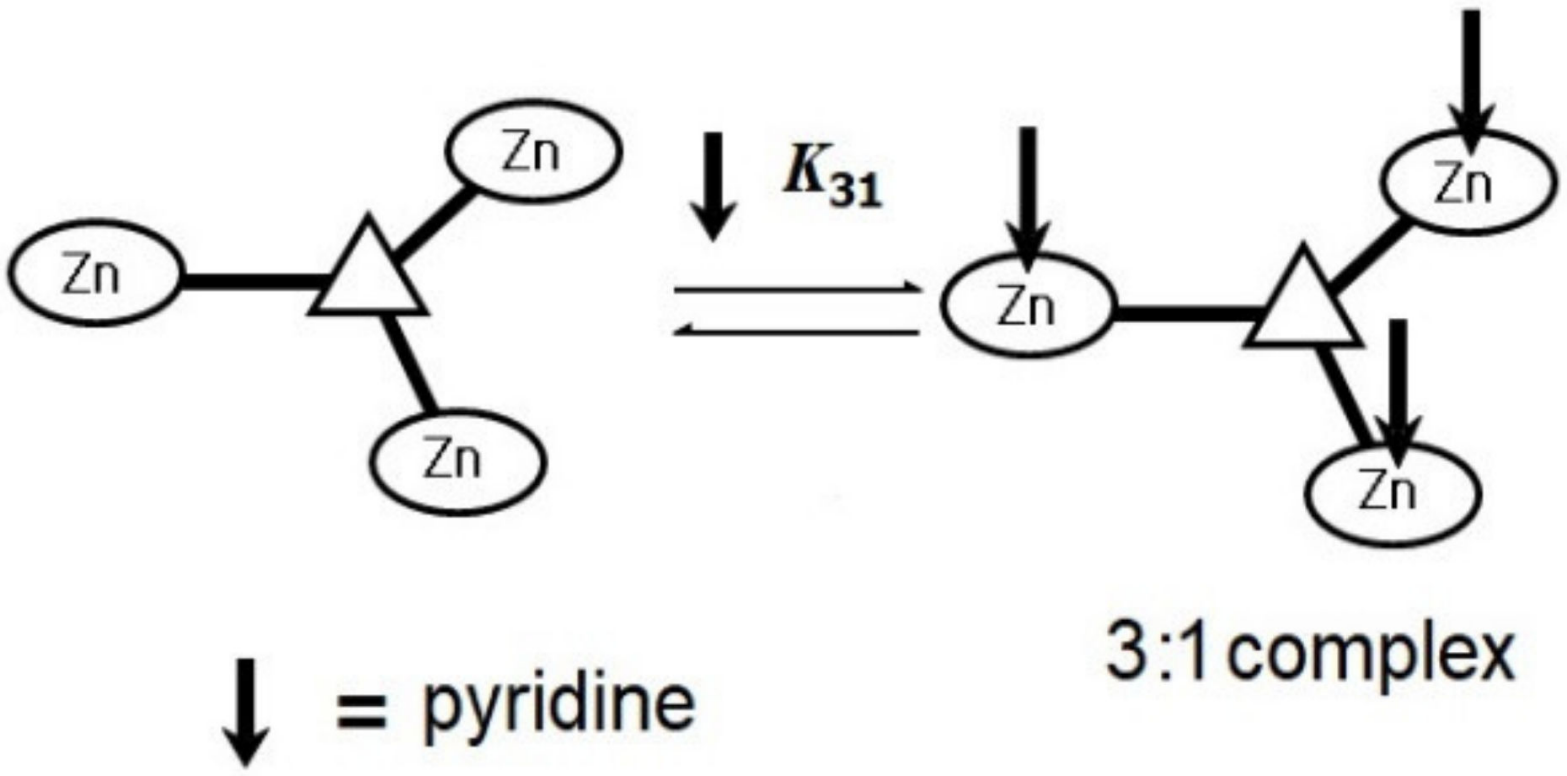
Schematic representation of the species involved in the binding of Zn-trisporphyrin **1** with pyridine.

**Figure 1:**
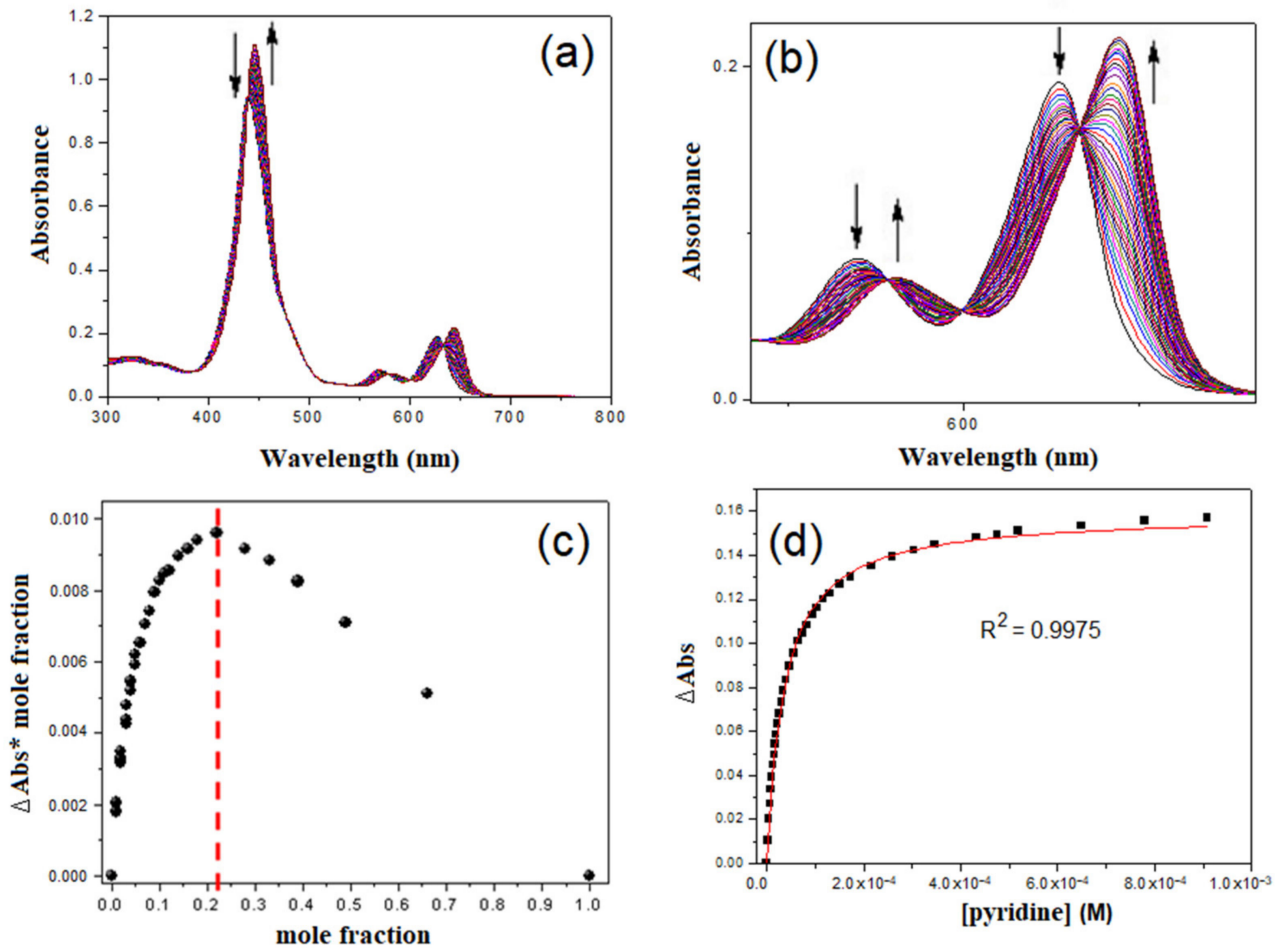
UV-vis titration spectra of Zn-trisporphyrin **1** complexation with pyridine in toluene (*c* = 1 μM). (**a**) UV-visible region, (**b**) Q-band zone, (**c**) data curve for Job’s plot at 446 nm (mole fraction of **1**), (**d**) fitting curve for the determination of the binding constant *K*_31_ at 446 nm.

The stability constant and the related species resulting from the coordination of pyridine with **1** are shown in Scheme 2. The UV-vis spectrum of **1** in toluene contained an intense, broad Soret band (439 nm) and weak Q-bands (570 nm and 627 nm) (Figure 1 and Supplementary Materials Figure S1) [34].

Titrimetric addition of pyridine to a toluene solution of **1** created a series of isosbestic points (439, 578, 599, and 632 nm) along with a shift in the Soret band (439 to 446 nm) and in the Q-bands (570 to 581 nm and 627 to 644 nm). The associated 7 nm redshift in the Soret band indicated pyridine binding to **1**. No further changes in the absorbance were observed after the addition of 3 equiv. of pyridine. Job’s plot for the titration data (Figure 1c) clearly supports forming a 3:1 complex, as shown in Scheme 2. Equilibrium constant *K*_31_ for **1**∙pyridine was calculated as follows:

(1) *K*_31_ = [(PY)_3_∙**1**]/[PY]^3^[**1**]

The binding constant, ***K*_31_** for the **1**∙pyridine complex, was estimated to be (2.7 ± 0.15) × 10^14^ M^−3^. On the other hand, monomeric Zn-porphyrin **2** exhibits typical electronic absorption features in toluene, including an intense Soret band at 434 nm and weak Q bands at 563 nm and 605 nm (Figure S2). The addition of pyridine to a toluene solution of monomeric **2** resulted in a series of isosbestic points (436, 570, 592, and 611 nm) along with a shift in the Soret band (434 to 438 nm) and in the Q-bands (563 to 575 nm and 605 to 622 nm). The associated 4 nm redshift in the Soret band is characteristic of the formation of a **2**∙pyridine complex in a 1:1 ratio. The binding constant, *K*_11_ (= [PY∙**2**]/[PY][2]) for the **2**∙pyridine complex, was calculated to be (1.60 ± 0.08) × 10^4^ M^−1^ (Figure S2).

**Scheme 3:**
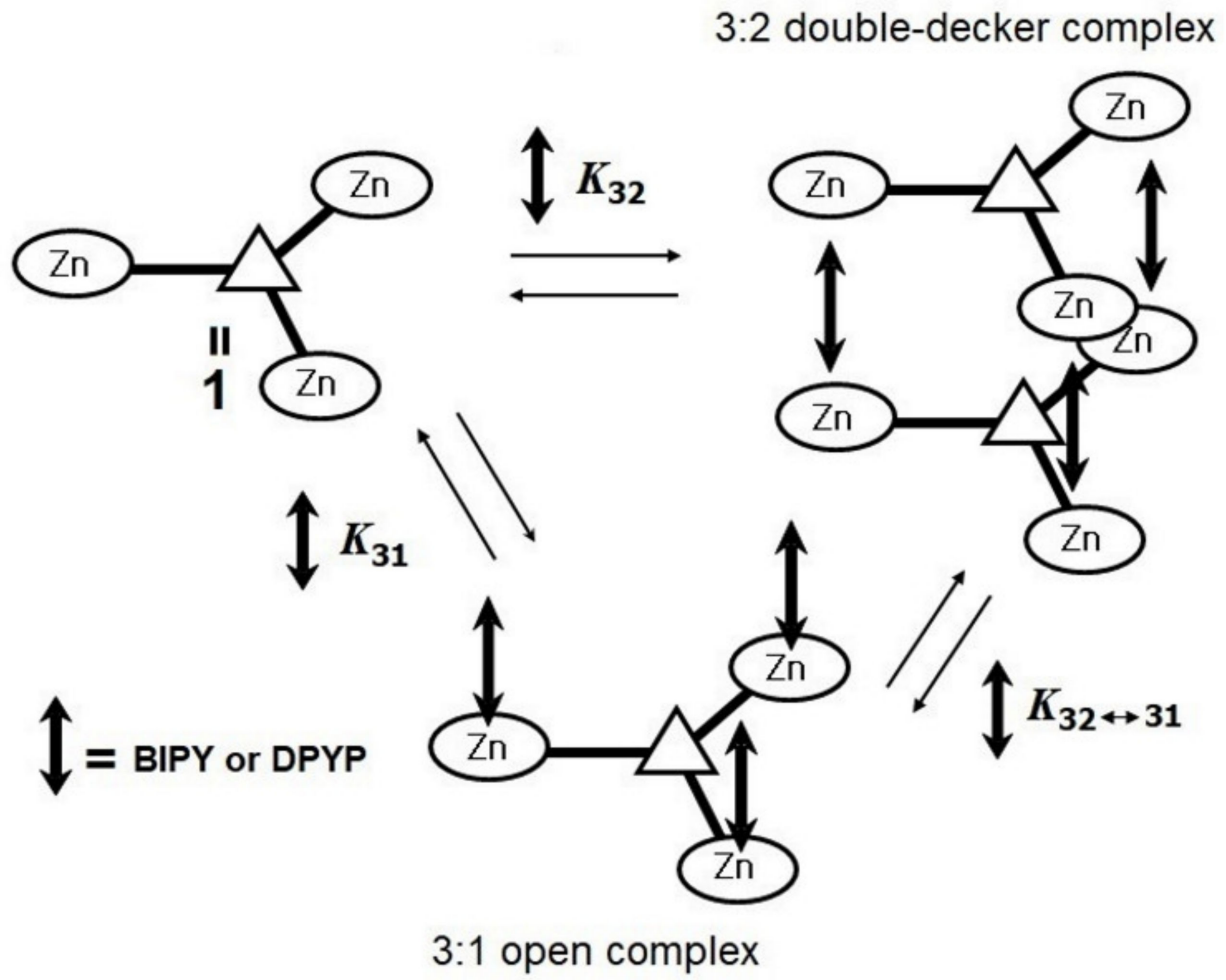
Schematic representation of the species involved in the binding of Zn-trisporphyrin **1** with BIPY or DPYP.

The addition of 4,4-bipyridine (BIPY) to a toluene solution of conjugated Zn-trisporphyrin **1** led to forming a series of coordination complexes. The overall stability constants and associated species are depicted in Scheme 3. The general equilibrium constants, shown in Scheme 3, were determined as follows:

(2) $$K_{31}={\left[L_{3}\cdot 1]/[L\right]}^{3}\left[1\right]$$

(3) $$K_{32}={\left[L_{3}\cdot 1_{2}]/[L\right]}^{3}{\left[1\right]}^{2}$$

(4) $$K_{32↔31}={\left(K_{31}\right)}^{2}/\left(K_{32}\right)={\left[L_{3}\cdot 1\right]}^{2}/\left[L_{3}\cdot 1_{2}\right]{\left[L\right]}^{3}$$

where, L = BIPY or DPYP.

**Figure 2:**
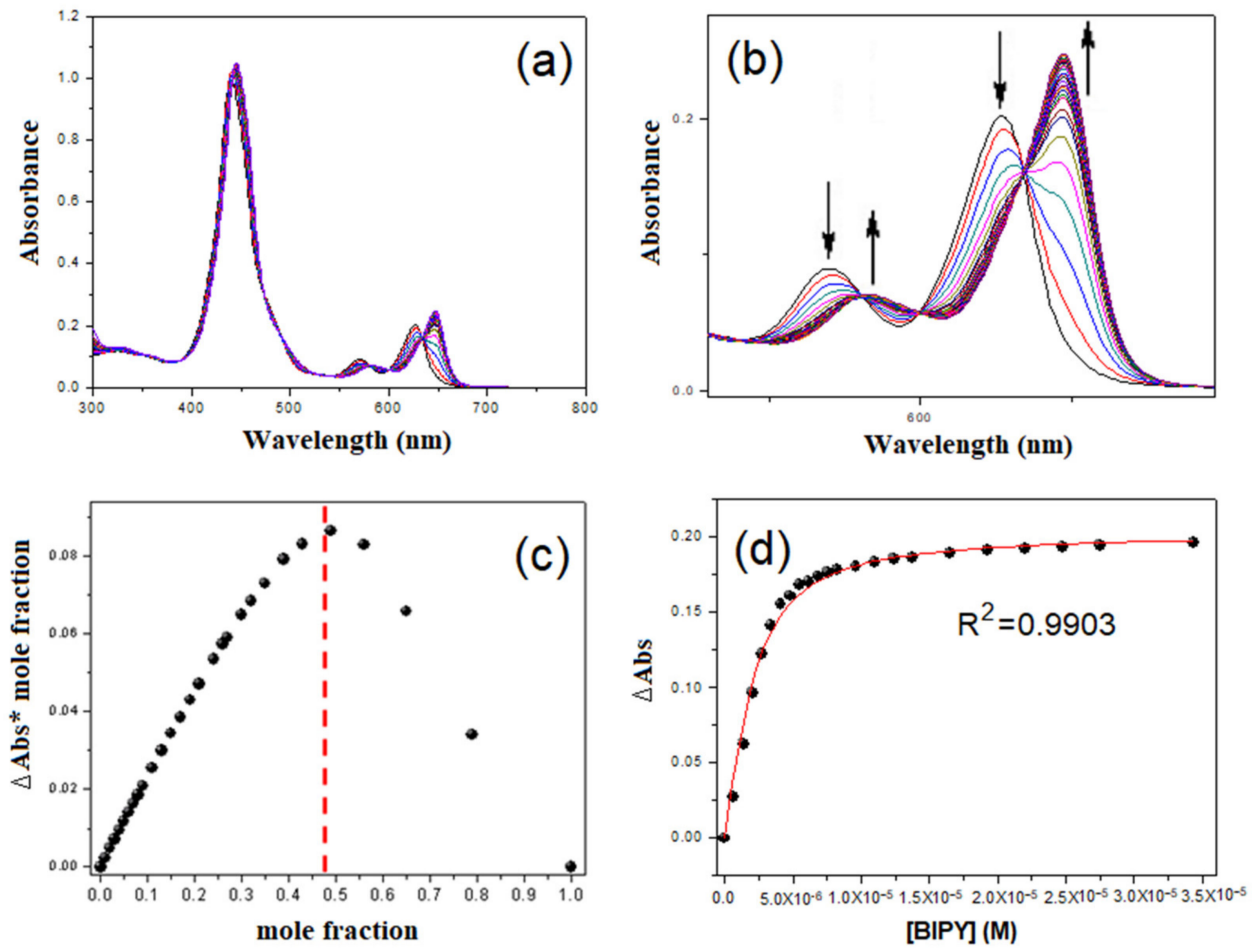
UV-vis titration spectra of Zn-trisporphyrin **1** with BIPY in toluene (*c* = 1 μM). (**a**) UV-vis region, (**b**) Q-band zone, (**c**) data curve for Job’s plot at 446 nm (mole fraction of **1**), (**d**) fitting curve for the determination of the binding constant *K*_32_ at 446 nm.

Upon the addition of 0–3 equiv. of BIPY into a toluene solution of **1**, the Soret band initially shifted from 439 to 443 nm (Figure 2 and Figure S3). Such a 4 nm redshift was characteristic of the formation of a 3:2 double-decker complex, as shown in Scheme 3. With the further addition of BIPY above 3 equiv. to the solution of **1**, the band at 443 nm started to decrease in intensity, and a new band appeared at 446 nm, suggesting the deconstruction of the 3:2 double-decker complex and the mono-coordination of **1** with BIPY to form a 3:1 open complex. In addition, a series of isosbestic points (441, 581, 599, and 634 nm) along with a shift in the Q-bands (570 to 583 nm and 627 to 647 nm) were also observed during the titration process. Based on the fitting of the titration data to the three species (**1**, 3:2 double-decker coordination complex, and 3:1 open complex) and the binding model described in Scheme 3, binding constants *K*_32_ and *K*_31_ were calculated to be (9.26 ± 0.07) × 10^27^ M^−4^ and (3.1 ± 0.13) × 10^15^ M^−3^, respectively, using global multivariate factor analysis [31,32,33,35,36,37]. On the other hand, the BIPY titration of a toluene solution of monomeric **2** resulted in a two-phase absorbance change, which appeared stable even after the addition of more than 3 equiv. of BIPY. The titration results consisted of four concomitant isosbestic points (436, 570, 592, and 611 nm) along with a shift in the Soret band (434 to 438 nm) and in the Q-bands (563–575 nm and 605–622 nm). The associated 4 nm redshift in the Soret band evidently supports forming a 1:1 **2**∙BIPY complex. From the titration data, it is clear that forming a 2:1 complex of Zn-monomeric **2** with BIPY was negligible under our experimental conditions. It is likely that 2:2 or 4:2 complexes were generated, which would affect the binding constant, as mentioned in the literature [32]. However, we did not consider these species owing to their negligible concentration under our experimental conditions (micromolar concentrations and room temperature). At micromolar concentrations, ladder-type 2:1 complexes are not likely to be formed during UV-visible titration [38]. The binding constant, *K*_11_ (= [BIPY∙**2**]/[BIPY][2]) for **2**∙BIPY, was calculated to be (2.02 ± 0.03) × 10^4^ M^−1^ (Figure S4). The above observation confirmed that the synergistic effect of the three conjugated porphyrin moieties in **1** was operational during the titration process [26]. This cooperative effect facilitates constructing a 3:2 double-decker coordination complex at a certain concentration of BIPY in solution.

**Figure 3:**
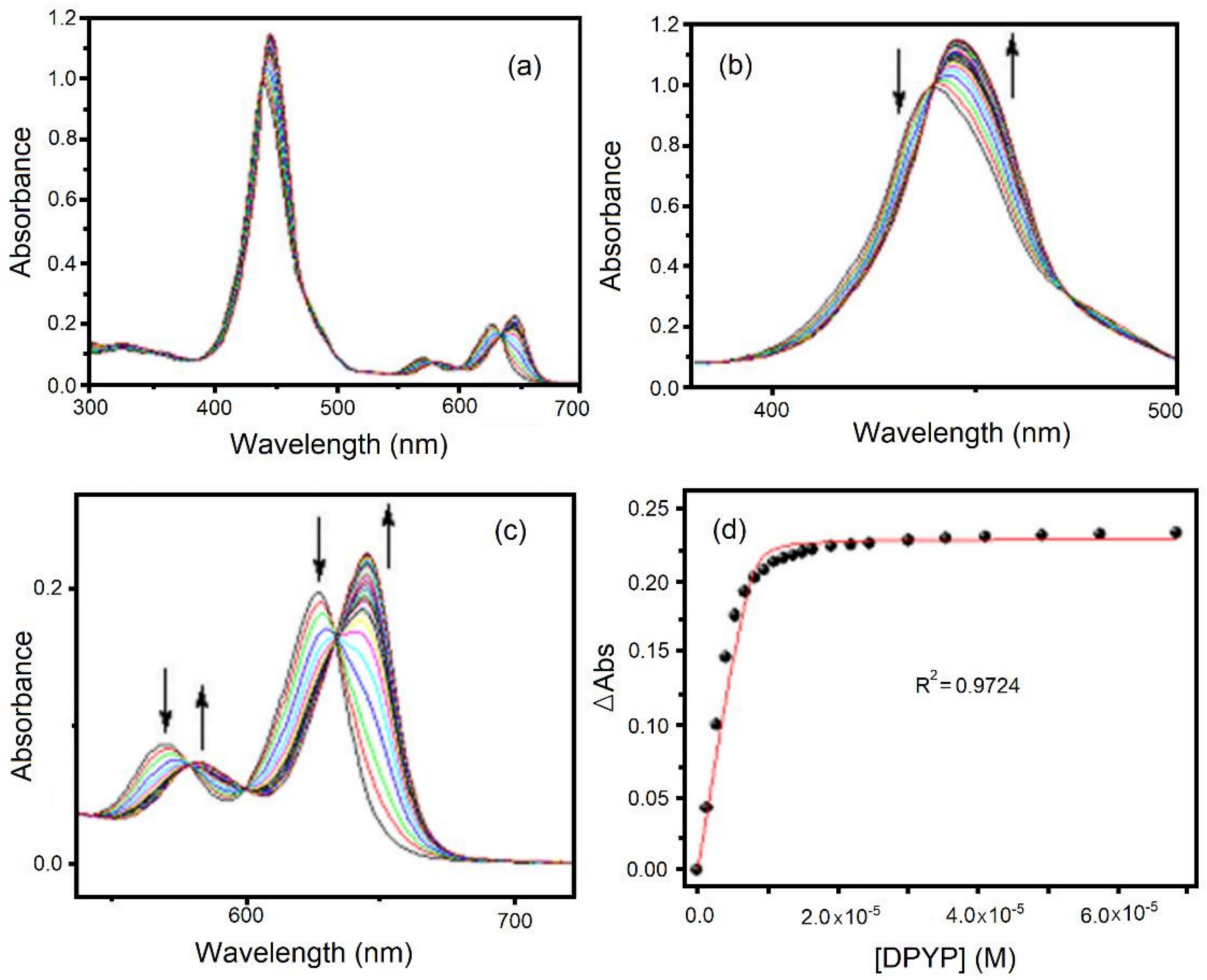
UV-vis titration spectra of Zn-trisporphyrin **1** with DPYP in toluene (*c* = 1 μM). (**a**) UV-visible region, (**b**) Soret-band zone, (**c**) Q-band zone, (**d**) fitting curve for the determination of the binding constant *K*_32_ at 446 nm.

**Figure 4:**
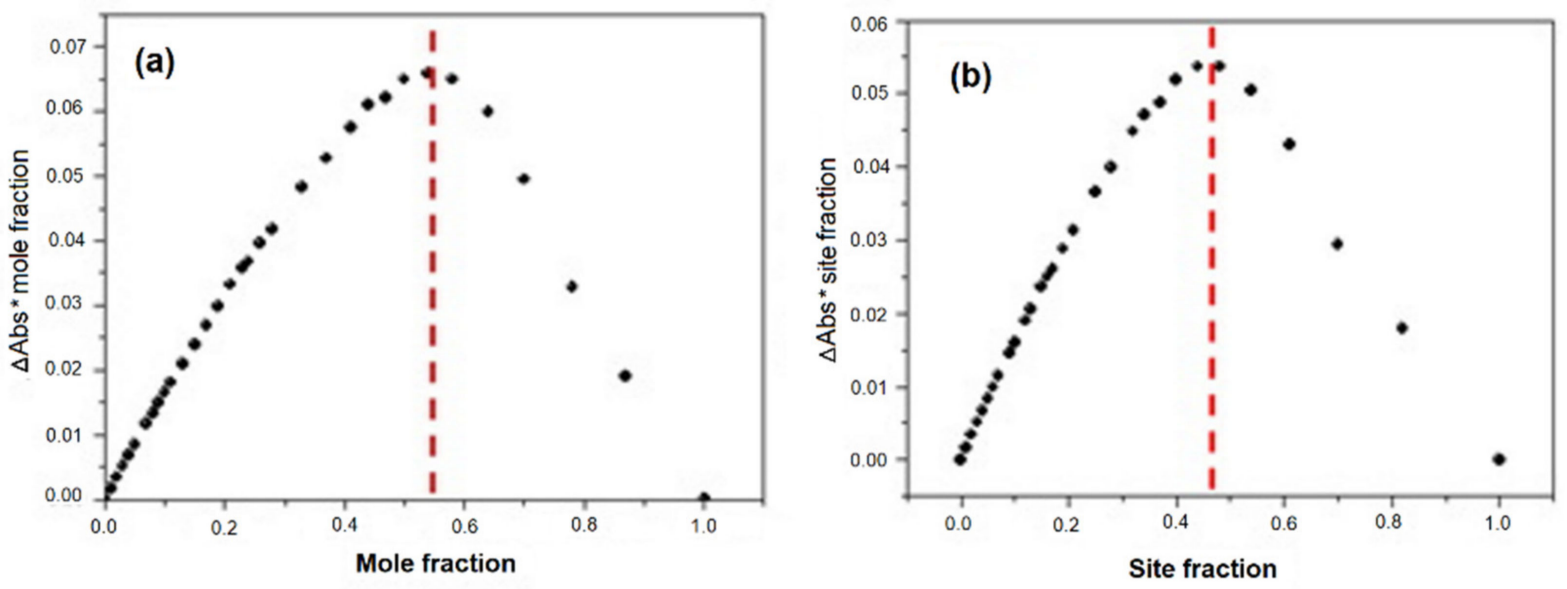
Job’s plots for the UV-vis titration of Zn-trisporphyrin **1** with DPYP in toluene (*c* = 1 μM). (**a**) mole fraction, and (**b**) site fraction.

The binding of the rigid ligand BIPY to **1** was then compared to the assembly behavior of **1** with flexible *N*-donor ligand, 1,3-di(4-pyridyl)propane (DPYP) in toluene. Titrimetric addition of DPYP to a toluene solution of **1** gave rise to a series of isosbestic points (439, 579, 599, and 633 nm) along with a shift in the Soret band (439 to 446 nm) and in the Q-bands (570 to 582 nm and 627 to 645 nm). The appearance of the titration profile was very similar to that of BIPY with **1** (Figure 3 and Figure 4), although the fitted curve did not perfectly match with experimental data. This deviation may be due to side reactions that form unexpected complexes.

**Figure 5:**
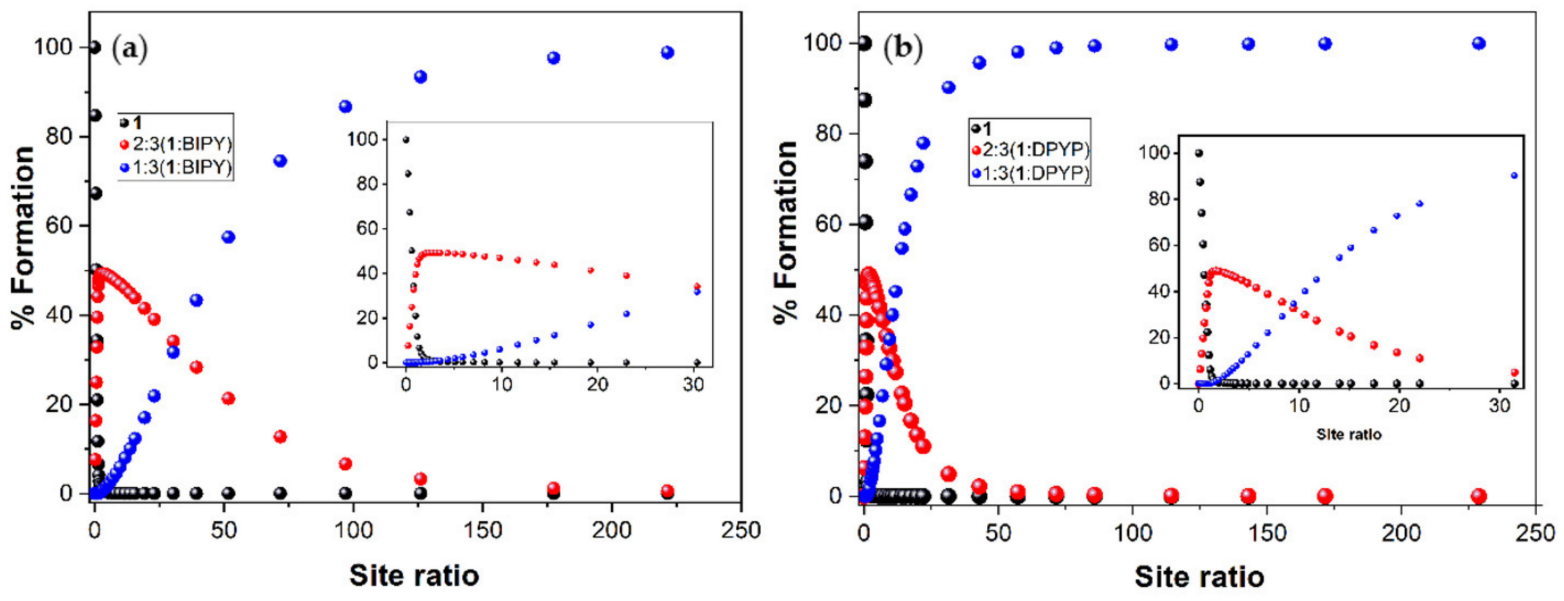
Simulated speciation profiles for titrations of **1** with (**a**) BIPY and (**b**) DPYP. Insets show enlargements of the lower-site-ratio region.

A notable difference is that the degradation of the sandwiched 3:2 complex and forming an open 3:1 complex proceeded more easily in the case of DPYP addition to the solution of **1** compared to that of BIPY titration. Binding constants *K*_32_ and *K*_31_ were calculated to be (3.62 ± 0.16) × 10^27^ M^−4^ and (8.1 ± 0.13) × 10^15^ M^−3^, respectively. Thus, the binding constant, *K*_32_ of **1**∙BIPY, was approximately 2.5 times higher than that of **1**∙DPYP. This implies that rigid BIPY forms a more robust self-assembled double-decker complex than flexible DPYP. Using the above stability constants of **1**∙BIPY and **1**∙DPYP, and the SPECFIT program, we simulated the speciation profile considering only three species (**1**, 3:2 sandwiched complex, and 3:1 open structure) according to the reported methods [32,33], as shown in Figure 5. It should be mentioned that for simplicity, we neglected other species (~2:2 or 4:2 model structures mentioned in [32]), due to their negligible content at micromolar concentration. We observed some notable features from these simulated speciation profiles along with the experimental spectra in Figure 2 and Figure 3. Initially, the concentration of **1** is high (100%), as indicated by the Soret band at 439 nm. After the addition of ligands (BPY or DPYP), the Soret band is redshifted to 443 nm, and the concentration of **1** is decreased. This is because, at a low concentration of ligand, both BIPY and DPYP formed 3:2 double-decker complexes with **1**. The concentration of the 3:2 double-decker complex increased upon the addition of bidentate ligands up to 3 equivalents. The 3:2 double-decker complex seems to be stable up to a certain range of ligand concentrations. After that, these double-decker complexes gradually degrade to form 3:1 open complexes as the concentration of ligands highly increases further. The Soret band was further redshifted from 443 nm to 446 nm during this process.

Several notable conclusions can be drawn from these UV-vis titration profiles. Degradation of the 3:2 double-decker **1**∙BIPY complex is less favorable compared to that of **1**∙DPYP. Consequently, forming the 3:1 open **1**∙DPYP complex proceeded more readily than that of **1**∙BIPY. It should be noted that DPYP is somewhat curved, whereas BIPY is a categorically linear ligand. Therefore, the possible geometry of the double-decker 3:2 **1**∙BIPY complex would be a regular sandwich (eclipsed stacked structure). On the other hand, the double-decker 3:2 **1**∙DPYP complex would exhibit offset sandwich geometry (displaced stacked structure).

To validate our results, we employed DABCO, a common bridging ligand, previously used to construct double-decker complexes [31,32,33,36]. Titrimetric addition of DABCO to a toluene solution of **1** afforded a series of isosbestic points (439, 578, 598, and 632 nm) along with a shift in the Soret band (439 to 446 nm), as well as in the Q-bands (570 to 582 nm and 627 to 645 nm). The appearance of the titration profile was comparable to that of BIPY titration of **1** (Figure S5). Binding constants *K*_32_ and *K*_31_ were calculated to be (1.47 ± 0.09) × 10^27^ M^−4^ and (1.7 ± 0.21) × 10^15^ M^−3^, respectively. The binding constant, *K*_32_ derived from **1**∙BIPY, was higher than that from **1**∙DABCO. Compared to DABCO, the longer arm of BIPY appears to avoid steric clashing with the aryl groups of **1** in forming double-decker complexes.

## 3. Materials and Methods

Zn-trisporphyrin conjugate **1** and monomeric Zn-porphyrin **2** were synthesized according to our previous report [34]. All other chemicals (pyridine, 4,4-bipyridine, and 1,3-di(4-pyridyl)propane) were purchased from Sigma-Aldrich and used without further purification. Steady-state UV-vis spectra were recorded on a Shimadzu UV-3600 spectrophotometer. For UV-vis titrations, we prepared the host and guest solutions in toluene at room temperature. The porphyrin (host) concentration was fixed at 1 μM, and ligand (guest) concentration was varied from 0.001 to 0.1 M. UV-vis titration experiments were performed by recording a spectrum of the host (porphyrin) in toluene at 298 K and adding the guest (ligand) solution in increments. After each addition, a new UV-vis spectrum was obtained. The data obtained from the UV-vis spectrophotometric titrations were analyzed by fitting the entire spectral series at 1 nm intervals using the SPECFIT software [39], as mentioned in earlier reports [32,33]. To determine the binding constants, the titration results were fitted using the nonlinear regression method within the Origin 9 software and generated the following equation:

(5) y = A((1 + *K*x + *K*P) − ((1 + *K*x + *K*P)^2^ − 4*K*^2P^x)^0.5^/2*K*P

x = [guest] × (binding site)

y = ΔA = A − A_0_

A =ΔA at 100% complexation

*K* = Binding constant

P = [host]

## 4. Conclusions

In summary, the supramolecular assembly of conjugated Zn-trisporphyrin **1** with different types of *N*-donor ligands was investigated in solution at micromolar concentrations, employing UV-vis spectrophotometric titration. While pyridine, a non-bridging ligand, forms a 3:1 open complex with **1**, bridging ligands, BIPY, and DPYP, form stable 3:2 double-decker complexes with **1**. These double-decker complexes collapse into simple open 3:1 complexes with the addition of excess BIPY or DPYP. For DPYP (flexible ligand) complexation with **1**, the 3:2 double-decker complex equilibrated with the 3:1 open complex more readily than the **1**∙BIPY complex (rigid ligand). Additionally, forming a 3:2 double-decker coordination complex was similarly observed when the DABCO complexed with **1**. Our results provide valuable insights for constructing a variety of metalloporphyrin-based self-assembled structures, potentially applicable in constructing novel functional materials for chemical detection, supramolecular catalysis, and photoelectronics.

## Data Availability

Data are available in the article and Supplementary Materials.

## Supplementary Materials

The following are available online. Figure S1: UV-vis titration spectra of Zn-trisporphyrin **1** with pyridine in toluene; Figure S2: UV-vis titration spectra of Zn-monoporphyrin **2** with pyridine in toluene; Figure S3: UV-vis titration spectra of Zn-trisporphyrin **1** with 4,4-bipyridine in toluene; Figure S4: UV-vis titration spectra of Zn-monoporphyrin **2** with 4,4-bipyridine in toluene, Figure S5: UV-vis titration spectra of Zn-trisporphyrin **1** with DABCO in toluene.

## Sample Availability

Samples of the compounds are not available from the authors.
