# Supplementary material for: Spectrophotometric Study of Bridging N-Donor Ligand-Induced Supramolecular Assembly of Conjugated Zn-Trisporphyrin with a Triphenylamine Core
Source: Molecules. 2021 Aug 6;26(16):4771. doi: 10.3390/molecules26164771 (PMC8399777; doi:10.3390/molecules26164771)
Supplement: Supplementary file 1 [file molecules-26-04771-s001.zip › molecules-1297757-supplementary.pdf]

Supplementary Materials

Spectrophotometric Study of Bridging *N*-donor Ligand-induced Supramolecular Assembly of Conjugated Zn-trisporphyrin with a Triphenylamine Core

Nirmal K. Shee, Ju-Won Seo and Hee-Joon Kim*

Department of Applied Chemistry, Kumoh National Institute of Technology
Gumi 39177, Republic of Korea

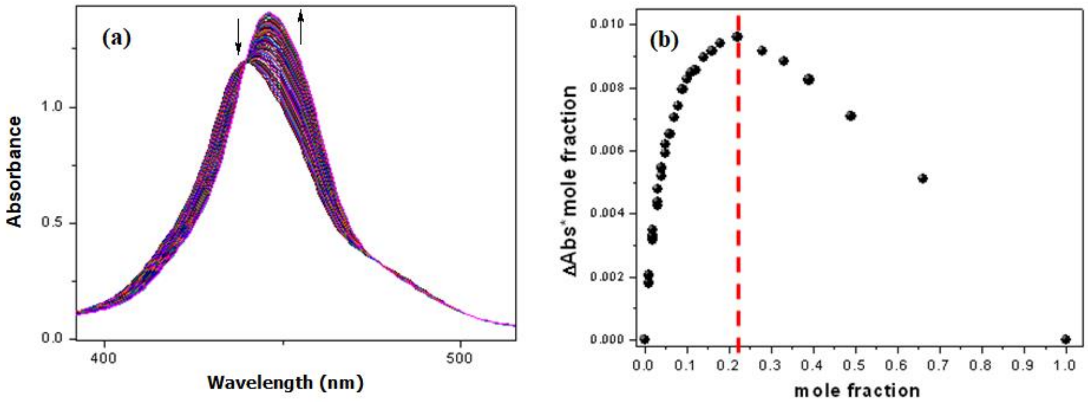

Figure S1. UV-vis titration spectra of Zn-trisporphyrin **1** with pyridine in toluene (c = 1 μM). (a) Soret band, and (b) data curve for Job's plot (mole fraction).

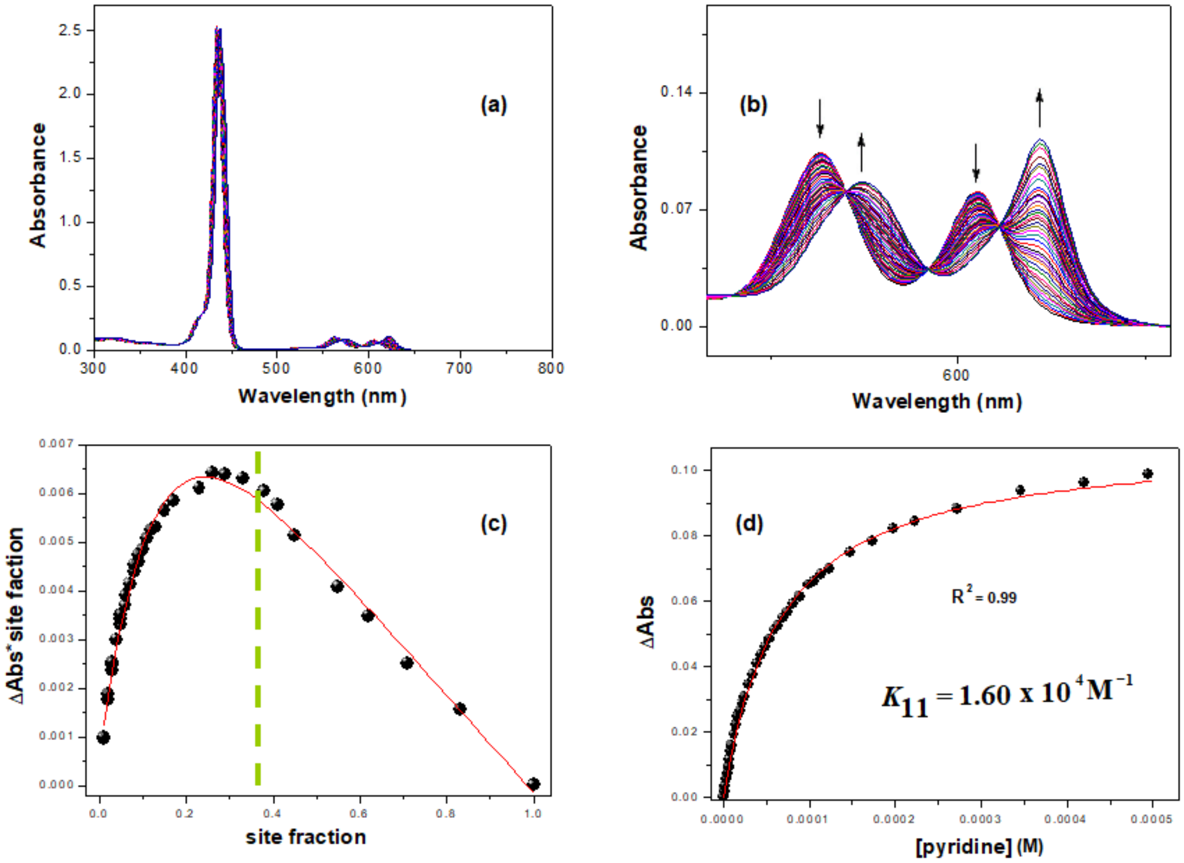

Figure S2. UV-vis titration spectra of Zn-monoporphyrin **2** with pyridine in toluene (c = 1 μM). (a) UV-visible region, (b) Q-bands zone, (c) data curve for Job's plot (site fraction), (d) fitting curve for the determination of binding constant $\boldsymbol{K_{11}}$.

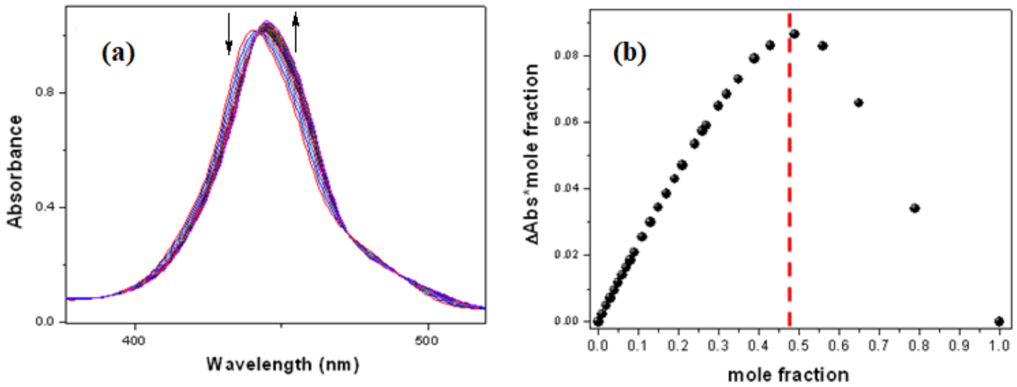

Figure S3. UV-vis titration spectra of Zn-trisporphyrin **1** with 4,4′-bipyridine in toluene (c = 1 μM). (a) Soret band, and (b) data curve for Job's plot (mole fraction).

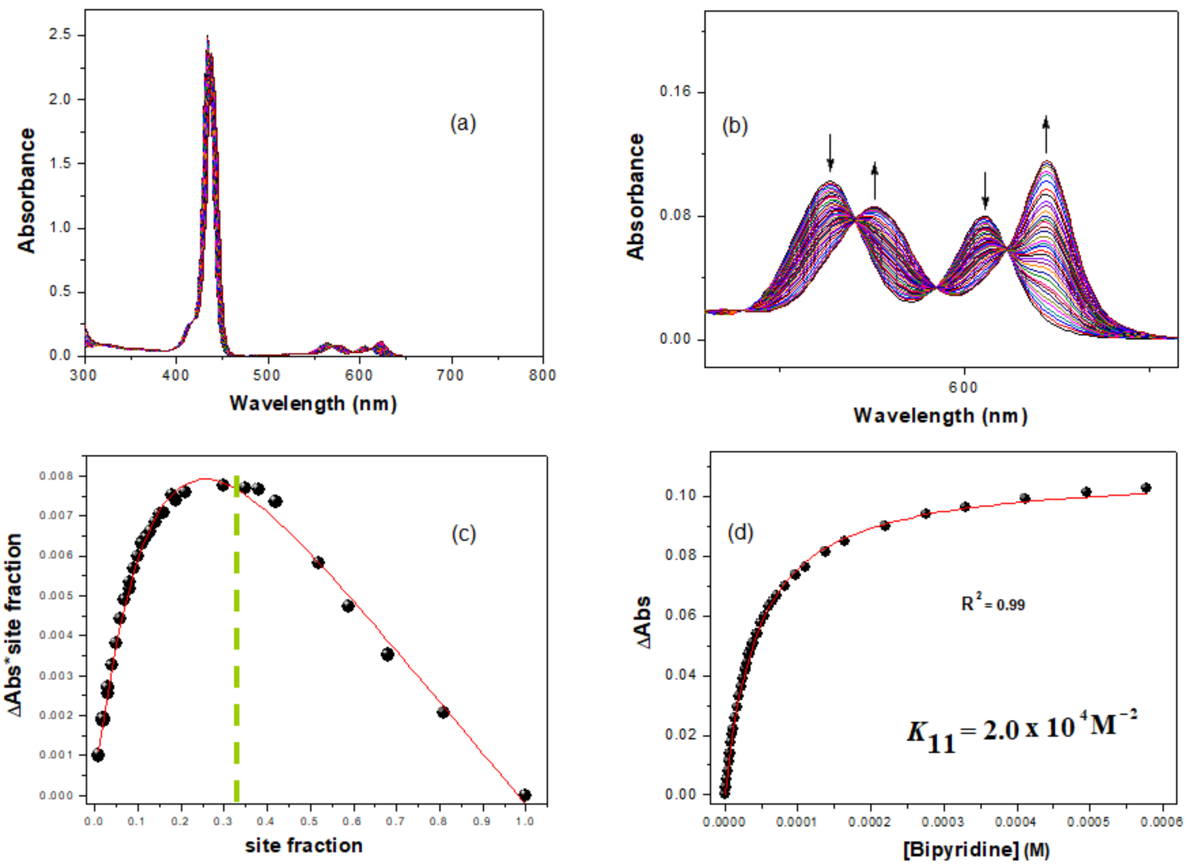

Figure S4. UV-vis titration spectra of Zn-monoporphyrin **2** with 4,4′-bipyridine in toluene (c = 1 μM). (a) UV-visible region, (b) Q-bands zone, (c) data curve for Job's plot (site fraction), (d) fitting curve for the determination of binding constant K_{11}.

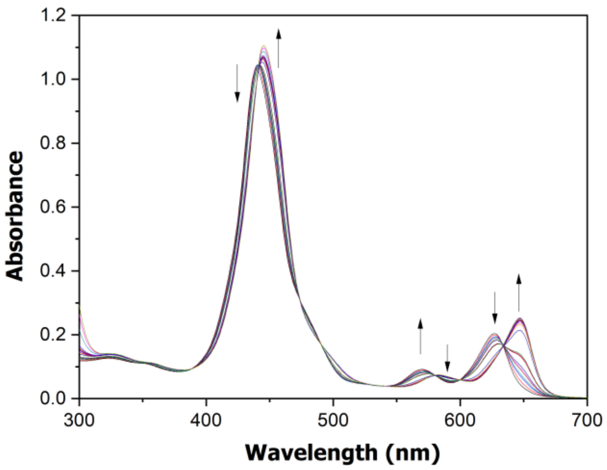

Figure S5. UV-vis titration spectra of Zn-trisporphyrin **1** with DABCO in toluene (c = 1 μM).
